# Adenosine Receptor 3 in Liver Cancer: Expression Variability, Epigenetic Modulation, and Enhanced Histone Deacetylase Inhibitor Effects

**DOI:** 10.1016/j.gastha.2024.11.006

**Published:** 2024-11-20

**Authors:** Louise Kaldjob-Heinrich, Sandro Nuciforo, Steffen Lemke, Aaron Stahl, Stefan Czemmel, Sepideh Babaei, Lauriane Blukacz, Marie-Anne Meier, Yizheng Zhang, Christian M. Schürch, Irene Gonzalez-Menendez, Pascal Woelffing, Nisar P. Malek, Veit Scheble, Sven Nahnsen, Manfred Claassen, Markus Templin, Hans Bösmüller, Markus H. Heim, Daniel Dauch, Michael Bitzer

**Affiliations:** 1 Department Internal Medicine I, Eberhard-Karls University, Tuebingen, Germany; 2 Department of Biomedicine, University Hospital and University of Basel, Basel, Switzerland; 3 Clinic of Gastroenterology and Hepatology, Clarunis University Center for Gastrointestinal and Liver Diseases Basel, Basel, Switzerland; 4 Quantitative Biology Center (QBiC), Eberhard-Karls University, Tuebingen, Germany; 5 M3-Research Center for Malignome, Metabolome and Microbiome, Eberhard-Karls University, Tuebingen, Germany; 6 NMI, Natural and Medical Sciences Institute at the University of Tuebingen, Reutlingen, Germany; 7 Department of Pathology and Neuropathology, Eberhard Karls University, Tübingen, Germany; 8 iFIT Cluster of Excellence EXC 2180 ‘Image-Guided and Functionally Instructed Tumor Therapies’, Eberhard-Karls University, Tuebingen, Germany; 9 Department of Medical Oncology and Pneumology, Eberhard-Karls University, Tuebingen, Germany; 10 Center for Personalized Medicine, Eberhard-Karls University, Tuebingen, Germany; 11 Department of Computer Science, University of Tübingen, Tübingen, Germany; 12 Machine Learning in Science, Excellence Cluster Machine Learning, University of Tübingen, Tübingen, Germany

**Keywords:** Adenosine Receptor 3, Hepatocellular Carcinoma, Cholangiocarcinoma, Epigenetic, Cancer Combination Therapy

## Abstract

**Background and Aims:**

Primary liver cancer, including hepatocellular carcinoma (HCC) and cholangiocarcinoma (CCA), has low response rates to existing treatments, highlighting the urgent need for novel treatment options. Adenosine A3 receptor (ADORA3) signaling has emerged as a potential target. Namodenoson, an ADORA3 agonist, has shown promise in early clinical trials for HCC. However, further data are required to clarify ADORA3 expression patterns in liver cancer, mechanisms of action, and the potential for combination therapies to inform patient selection for future clinical trials.

**Methods:**

Patient-derived tissue microarrays and RNA-sequencing were employed to investigate ADORA3 expression. Cellular responses to ADORA3 stimulation and combination treatments were studied in HCC and CCA cell lines and patient-derived organoids (PDOs). Genome-wide RNA-Seq analysis, mRNA analysis, and DigiWest protein profiling were performed.

**Results:**

Tissue microarray analysis revealed higher ADORA3 expression in nonmalignant samples and a subset of tumors with weak or absent ADORA3 expression. This was supported by RNA sequencing data from The Cancer Genome Atlas and needle biopsy samples. Cell lines and PDOs exhibited antiproliferative effects with the ADORA3 agonist Namodenoson, confirmed by receptor dependency tests with specific antagonists and siRNA experiments. Genome-wide RNA-Seq analysis suggested chromatin remodeling events after ADORA3 stimulation. mRNA expression and DigiWest profiling identified downregulation of histone deacetylases and histone H3 modifications. Combination treatments with different ADORA3 agonists and histone deacetylase inhibitors significantly enhanced antiproliferative effects in almost all selected combinations, supported by investigations in PDOs.

**Conclusion:**

ADORA3 expression varies considerably in HCC or CCA, ranging from high to absent receptor detection. This observation might help to identify patients for clinical studies. Additionally, Namodenoson’s epigenetic modulating activity suggests epigenetic drugs as promising candidates for combination treatment.

## Graphical abstract

**Figure:**
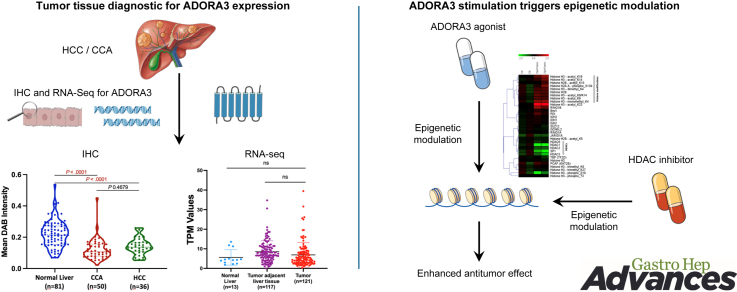

## Introduction

Primary liver cancer is a major global health problem, with about 800,000 patients annually dying of this disease.^1^ Systemic treatment options for advanced disease stages exist for hepatocellular carcinoma (HCC) and cholangiocarcinoma (CCA). However, despite the growing number of available drugs, the objective response rates for HCC or CCA are reported between 26% and 36%.^2^, ^3^, ^4^, ^5^, ^6^, ^7^ These numbers show that a substantial fraction of patients still do not respond to available treatments. Therefore, a high unmet need exists to identify new treatment options that address a different action mode. In this context, modulation of adenosine receptor (ADORA) signaling by stimulation of purinergic ADORAs has been suggested as a potential new target in liver cancer.^8^^,^^9^

Adenosine is a ubiquitous signaling molecule that binds to 4 subtypes of ADORAs, ADORA1, ADORA2A, ADORA2B, and ADORA3. These receptors’ involvement has been widely studied in pathological conditions such as tissue protection, liver fibrosis, anti-inflammation, or cancer.^10^, ^11^, ^12^ ADORA signaling is an essential factor in tumor biology that affects both the immune system and intracellular signaling cascades in tumor cells.^13^^,^^14^ Interestingly, muscle cells secrete natural agonists to ADORA3 with an antitumor effect that seems to account for the rarity of tumor metastases in striated muscles.^15^ The gene encoding for ADORA3 is widely expressed in various tissues, including the brain, heart, lung, and liver. This receptor has been extensively investigated as a potential drug target during the last decade.^9^^,^^16^, ^17^, ^18^, ^19^

In normal hepatocytes, ADORA3 stimulation with the specific agonist Namodenoson (CF102) accelerates mitosis with an improved regeneration after partial hepatectomy.^20^ In contrast, hepatoma cells show an opposite regulation pattern with inhibition of proliferation and upregulation of proapoptotic factors,^21^ or modulation of cancer signaling pathways, such as ERK1/2, Akt,^19^ β-Catenin, or c-Myc.^8^ ADORA3 has been described to be upregulated in tumor tissues from small groups of patients with mesothelioma,^22^ thyroid,^23^ breast and colon cancer.^24^^,^^25^ A detailed characterization of ADORA3 expression in HCC or CCA tumor samples has not been reported yet. The assumption of ADORA3 overexpression in liver tumors has been based on observations in other tumors, single cases of mRNA expression in HCC tissues, or ADORA3 levels in peripheral blood mononuclear cells, which were postulated to be a surrogate marker for tumor expression.^8^^,^^26^ However, data on protein expression in patient-derived HCC samples or data on ADORA3 in CCA have not been reported yet.

The stimulation of ADORA3 by Namodenoson has already reached clinical testing for metabolic dysfunction-associated steatohepatitis (MASH)^27^ and HCC.^26^^,^^28^ The HCC trial investigated the drug in a phase II study with 78 patients with Child-Pugh B liver function and advanced tumors.^26^ At first glance, this study did not reach its primary endpoint of a statistically significant overall survival difference. Despite this negative result, Namodenoson led to an objective response in single patients and to a 12-month survival rate of 44% of patients with Child-Pugh B7, compared to 18% in the placebo group.^26^ These early clinical results show that stimulation of ADORA 3 in primary liver cancer is feasible and safe, and further suggest that a subgroup of patients might benefit from that treatment.^29^ However, to select relevant patient groups in further clinical trials, more data are needed to characterize ADORA3 and cellular responses after receptor stimulation in liver cancer.

Here, we report a wide variety of ADORA3 expression as a new finding in patient-derived HCC and CCA tissues, including tumors with high receptor detection and others entirely missing it. Regarding the role of ADORA3 as a potential drug target, antiproliferative effects were dependent on drug-mediated receptor stimulation. Moreover, as a new mode of action, we discovered Namodenoson to harbor epigenetic modulating activity, with a broad inhibition of enzymes with histone deacetylase (HDAC) activity in cancer cells. Different HDAC inhibitors (HDACis) could increase the therapeutic efficacy of ADORA3-stimulating drugs. These observations are relevant for the further clinical development of ADORA3 activation as a therapeutic principle in liver cancer. On the one hand, ADORA3 expression levels on tumor cells should be analyzed before study entry. On the other hand, epigenetic drugs, such as HDACis, are attractive new candidates for combination treatments.

## Materials and Methods

### Cell Culture and Human HCC and CCA Organoid Cultures

Human hepatoma cell lines included HepG2, Huh7, and TFK1 cells, human CCA cell lines HUCCT1, and RBE cells. All patient-derived organoids (PDOs) were generated and established as previously described.^30^

### Tissue Microarrays (TMAs)

Tissue microarray (TMA) from paraffin blocks were retrieved from the Institute of Pathology archives at the University of Tübingen as described.^31^ The analysis included samples from 36 patients with HCC and 51 patients with intrahepatic cholangiocarcinoma and was approved by the Ethics committee of the Medical Faculty at Tübingen University (880/2020BO2). ADORA3 immunohistochemistry and quantification is described in Supplementary Materials and Methods.

### RNA-Seq Analysis and Bioinformatics

Transcriptome analysis of HepG2 cells was performed after total RNA extraction and purity control. Sequencing was performed by using one SP Flow-Cell (725 Mio Clusters, 50 BP, paired ended) on an Illumina Novaseq machine at the NCCT at the University of Tuebingen. Bioinformatics and differential expression analysis were performed in cooperation with the Quantitative Biology Center at the University of Tuebingen. RNAseq data of HCC needle biopsies were provided by Ng et al.^32^

### The Cancer Genome Atlas Liver Hepatocellular Carcinoma (TCGA-LIHC) RNA-Seq Tissue Biopsy Data

Through the recount2 project portal, gene counts (version 2) of The Cancer Genome Atlas Liver Hepatocellular Carcinoma (TCGA-LIHC) cohort were obtained (https://jhubiostatistics.shinyapps.io/recount/).^33^ Samples from patients were used only when both HCC and adjacent healthy liver tissue samples were available. In total, 50 HCC samples and 50 adjacent healthy tissue samples were included in the analysis.

### ADORA3 scRNA-Seq Expression

Publicly available raw count data from a recent single-cell RNA sequencing study in HCC^34^ was downloaded from the Gene Expression Omnibus database under the accession code GSE149614.

### DigiWest Multiplex Protein Analysis

DigiWest protein profiling was performed as described^35^ using 12 μg of quantified cell lysates per sample. An exact description of the DigiWest procedure is included in Supplmentary Materials & Methods and the antibody list is shown in Table A4C.

### Synergy Screening and Drug Synergy Analysis with Organoids

Namodenoson and HDACis combination screens were performed in 5 × 6 dose-response matrices. All organoids were treated with 0 (Dimethylsulfoxid (DMSO), solvent control) up to 20 μM Namodenoson, 0 (DMSO, solvent control) up to 5 μM Resminostat, Belinostat or Vorinostat and 0 (DMSO, solvent control) up to 5 nM Romidepsin. Drug synergy scores were generated using the online version of SynergyFinder 2.0.^36^^,^^37^ The zero interaction potency model was used and the average synergy score over the landscape as well as the area of the highest synergistic interaction were calculated as previously described.^36^^,^^38^ A synergy score lower than −10 is considered an antagonistic combination, between −10 and 10 is considered additive and above 10 synergistic.

All research was conducted in accordance with both the Declarations of Helsinki and Istanbul, all research was approved by the appropriate ethics or institutional review committees if human-derived samples were used. For further details regarding materials and methods, please refer to Supplementary Materials & Methods.

## Results

### Heterogeneous Distribution of ADORA3 in HCC and CCA Tissues

**Figure 1:**
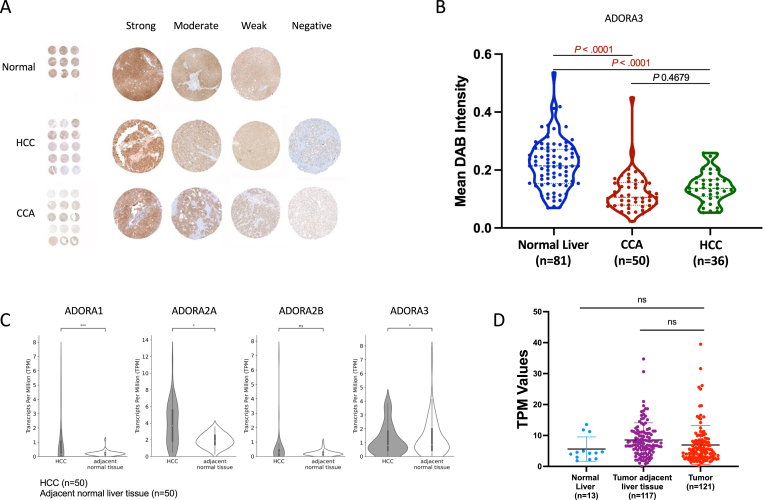
Heterogeneous level of ADORA3 in HCC and CCA tissues. (A) TMAs from paraffin blocks of patients with HCC and CCA were stained for ADORA3 by immunohistochemistry. For each case, a total of 3 cores representing tumor tissue and 3 cores with normal liver tissue were investigated. The detection of ADORA3 was analyzed per high power field (magnification ×400), and for the semiquantitative evaluation, a score between 0 (no ADORA3 detectable, negative) and 3 (intense ADORA3 signal, strong) was attributed to each tissue core. Shown are examples for each staining result. (B) ADORA3 immunostainings of TMAs for normal liver (*n* = 81, blue dots), CCA (*n* = 50, red dots) and HCC (n = 36, green dots) tissue samples. Data represent the average of triplicate semi-quantative scores for ADORA3 staining. (C) RNA-Seq tissue biopsy data from the TCGA-LIHC cohort. The Box plots represent gene expression for ADORA1, ADORA2A, ADORA2B, and ADORA3 in HCC (n = 50) and healthy liver tissue (n = 50), derived from patients from whom both tumor and adjacent normal tissue were available. Gene expression data are shown as transcript per million. Asterisks indicate a statistically significant difference (∗*P* < .05, ∗∗∗*P* < .001, ns, not significant) obtained from differential expression analysis using DESeq2. (D) ADORA3 expression in patient-derived needle biopsies from normal liver (n = 13), tumor adjacent liver tissue (n = 117), and HCCs (n = 121). Black bars represent the mean ± stable disease. Gene expression data are shown as transcript per kilobase million. Statistical analysis was performed using 1-way ANOVA (and nonparametric) with Dunn’s multiple comparisons test (ns, not significant).

The reports of 2 phase II clinical trials that investigated Namodenoson in patients with MASH^27^ or HCC^26^ were based on the assumption that ADORA3 should be overexpressed in inflammatory or tumor tissues compared to healthy tissues. These assumptions were part of the central hypotheses of these trials. However, a systematic investigation of patient or study participants’ tissue samples has not been reported yet. Therefore, we investigated the receptor level in normal liver, HCC, and CCA tissue by immunohistochemistry using patient-derived TMAs. In a first step, a semiquantitative analysis comparing triplicates differentiated between “strong,” “moderate,” “weak,” and “negative” revealed conflicting results to this previous hypothesis (Figure 1A). To our surprise, most of the normal 81 liver samples revealed a “strong” receptor level, and no liver tissue was graded as “negative.” In more detail, we next analyzed the TMA sample staining intensity per pixel. Of note, the staining of normal tissues revealed significant higher ADORA3 levels compared to CCA or HCC samples. Moreover, several samples in both tumor types showed hardly any detectable ADORA3 staining (Figure 1B).

To investigate this finding further, we examined gene expression of all 4 ADORA receptors in human RNA-Seq tissue biopsy data from the publicly available data of the TCGA-LIHC cohort. In the analysis, we included 50 patients of the cohort from whom both tumor and adjacent normal data were available. Interestingly, this analysis revealed an overexpression of ADORA1 and 2A in HCC tissues, but no difference for ADORA2B. In contrast, the expression of ADORA3 in the adjacent normal liver tissue was slightly higher than in HCCs (Figure 1C), which was in concordance with the TMA analysis. Additionally, analyzing RNA sequencing data from patient-derived needle biopsy samples, we compared *ADORA3* expression between normal liver tissue, tumor-adjacent liver tissue, and HCC tumors. As a result, we could confirm a similar expression pattern of ADORA3 in tumors of HCC, which was not statistically different from normal liver or tumor-adjacent tissues (Figure 1D). Taken together, our results show a heterogeneous expression of ADORA3 in liver tumors, including tumors with high and others with missing ADORA3 expression.

### ADORA3 Mediates Antiproliferative Effects of Namodenoson in HCC- and CCA-Derived Established Cell Lines and PDOs

**Figure 2:**
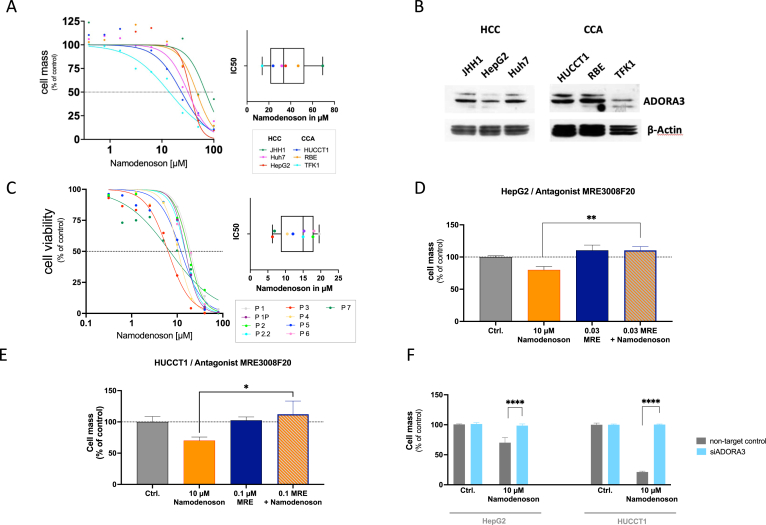
ADORA3 mediates antiproliferative effects of Namodenoson in HCC- and CCA-derived cell lines and human organoids. (A) Dose-dependent drug-induced cytotoxicities of Namodenoson on established human-derived HCC (HepG2, Huh7, JHH1) and CCA (HUCCT1, RBE, TFK1) cell lines. The cytotoxicity was determined by SRB assay and plotted as a function of cell mass. The Box-Plot summarizes all measured IC50 values including the median. (B) Representative western blot analysis of ADORA3 in different HCC- (JHH1, HepG2, Huh7) and CCA- (HUCCT1, RBE, TFK1) derived cell lines. (C) Dose-dependent antiproliferative effect of Namodenoson in HCC and CCA patient-derived organoids. Cell viability was determined by cell titer glow assay after 4 days of treatment. Box-Plot summarizes all IC50 values and shows the median of the Namodenoson mediated effect in all used organoids (right). (D + E) ADORA3 antagonist MRE3008F20 (MRE) opposes the antiproliferative effect of Namodenoson. HepG2 (C) and HUCCT1 (D) cells were incubated with MRE3008F20 before treatment with Namodenoson or solvent as control. The percentage of cell mass was determined 72 h later by SRB assay. Data represent standard error of the mean of at least 2 independent experiments performed in duplicates (∗*P* < .05; ∗∗*P* .01). (F) ADORA3-siRNA blocks the antiproliferative effect of Namodenoson. HepG2 and HUCCT1 cells were transfected with siRNA against ADORA3 (*siA3R)* or scrambled control (Non-Target Ctrl) siRNAs. Forty-eight hours after transfection cells were treated with 10 μM Namodenoson or solvent as control. Cell mass was determined via SRB assay 72 hours later. Data represent standard error of the mean of at least 2 independent experiments performed in duplicates (∗∗∗∗*P* < .0001). SRB, Sulforhodamin B Cytotoxicity.

To characterize the influence of ADORA3 stimulating drugs on postulated antitumor effects, we next focused on antiproliferative properties in cancer cells. First, we investigated Namodenoson in several established human-derived cell lines. Sulforhodamin B Cytotoxicity assays were performed using the human hepatocyte-derived carcinoma cell lines HuH7, JHH1, and HepG2, and the biliary duct-derived carcinoma cell lines HUCCT1, RBE, and TFK1. All these cell lines showed increasing antiproliferative effects with rising concentrations of the ADORA3 agonist with IC50 values between 13,9 μM for TFK1 and 69,3 μM for JHH1 (Figure 2A, Table A1A). Western blot analysis and immunocytochemical staining demonstrated the presence of ADORA3 in all employed cell lines, albeit at different levels (Figures 2B and A1). We could not identify a clear correlation between the level of ADORA3 expression and sensitivity to Namodenoson. This might be attributed to the fact that receptor expression levels do not necessarily correlate directly with the intensity of the functional response to agonist stimulation, as cellular sensitivity to ADORA3 agonists can be modulated by a variety of factors, such as the degree of receptor coupling to effector pathways or different downstream signaling components. To expand the investigations in established cell lines to patient PDOs, we subsequently investigated HCC- (Pat. 1–6), and CCA-derived (Pat. 7) PDOs (Figure 2C, Table A1B). Of note, most of these organoids showed a higher sensitivity with a lower IC50 value compared to the established cell lines.

To verify that the antiproliferative effect of Namodenoson is indeed mediated via ADORA3, the two cell lines HepG2 (HCC cell line) and HUCCT1 (CCA cell line) were coincubated with MRE3008F20, a specific ADORA3 receptor antagonist. In both experiments, the antagonist could block the antiproliferative effect of 10 μM Namodenoson (Figure 2D and E). Furthermore, siRNA experiments with target RNA to ADORA3 in both cell lines efficiently prevented the antiproliferative effect of Namodenoson (Figure 2F). Taken together, these experiments demonstrated that ADORA3 activation is the major mechanism for the observed antiproliferative effect of Namodenoson.

### Namodenoson Alters Gene Expression in Epigenetic Pathways

**Figure 3:**
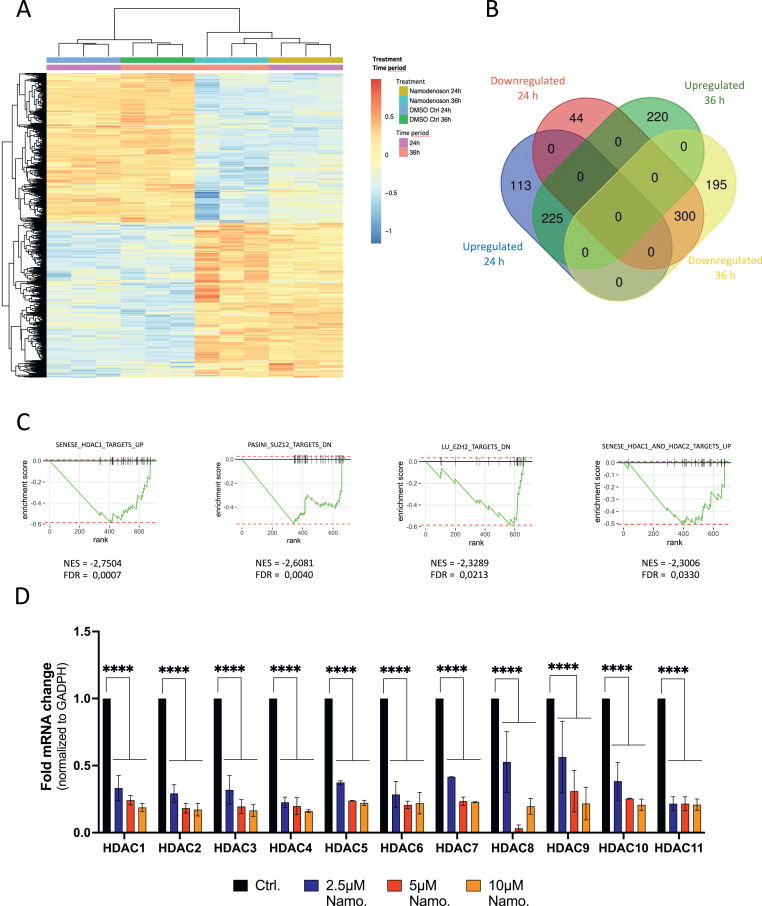
HepG2 RNA-Seq. analysis reveal profound changes in gene expression after treatment with Namodenoson. (A) Heatmap-based visualization of hierarchical clustering of the differential expressed genes (padj < 0.05 and log2FC < −0.5| > .5) found in RNA-Seq data. The dendrogram on the left and top illustrate the clustering euclidean distance. Sample groups and the distribution of the normalized expression values are shown as color keys on the top and top right, respectively. Note that these values were also scaled on rows to have mean 0 and standard deviation 1. Red indicates lower expression and blue indicates higher expression. (B) Overview of the number of unique and shared significantly deregulated genes after 24 (red and blue circles) and 36 hours (green and yellow circles) of Namodenoson treatment. (C) Gene set enrichment analysis of RNA-seq data after 24-hour treatment with Namodenoson. Enrichment plot showing downregulation of different gene sets associated with epigenetic regulation indicating false discovery rate, adjusted *P* value, and normalized enrichment score. (D) Real-time PCR analysis of HDAC 1–11 mRNA in HepG2 cells treated with rising concentrations of Namodenoson (2.5, 5, 10 μM) for 24 h. Data represent standard error of the mean of 3 independent experiments. Statistical analysis was performed using 2-way ANOVA with Tukey’s multiple comparisons test (∗*P* < .05; ∗∗*P* .01, ∗∗∗*P* .001, ∗∗∗∗*P* < .0001, ns, not significant).

Despite the application of Namodenoson in clinical trials to stimulate ADORA3, the mode of action in tumor cells of this drug is only partly understood. Therefore, we performed a genome-wide RNA-Seq analysis in HepG2 cells treated either for 24 or 36 hours with Namodenoson. Sample clustering using a principal component analysis and visualization of the resulting changes in the transcriptome using a heatmap showed a clear separation of both time points with Namodenoson compared to DMSO-treated control cells (Figures 3A and A2A). This observation implies a strong and unique gene expression pattern upon the stimulation of ADORA3. In more detail, of the 36,521 mapped gene tags in HepG2 cells, 682 genes (with 338 upregulated and 344 downregulated genes) and 940 genes (with 445 upregulated and 495 downregulated genes) were differentially expressed comparing Namodenoson treated vs control cells at 24 and 36 hours, respectively, with 525 of them shared (Figures 3B and A2B, Table A2).

**Figure 4:**
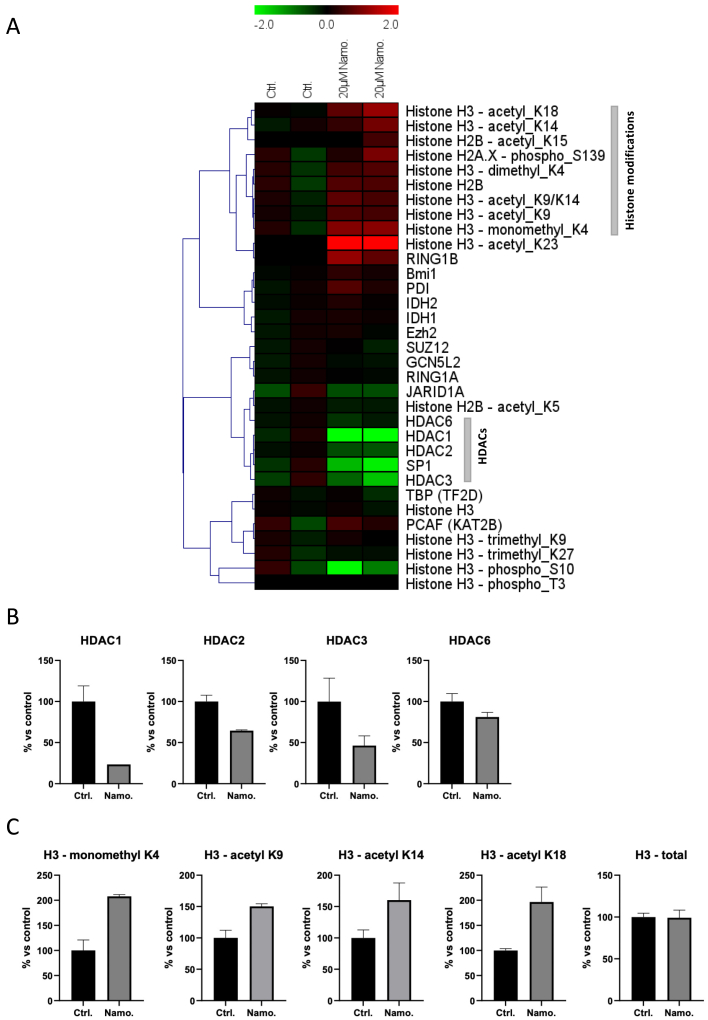
DigiWest protein profiling of Namodenoson-treated HepG2 cells. (A) Hierarchical cluster analysis of epigenetically relevant analytes. Expression values were normalized to total protein signal across all samples for a given analyte, set in relation to the average control signal and Log-2 transformed. Hierarchical cluster analysis was performed using Pearson Correlation and complete linkage. Most analytes were selected from the DigiWest antibody library based on GOTerms GO:0006325 (chromatin organization) and GO:0040029 (regulation of gene expression). (B) Expression values relative to control (in %) for HDACs. (C) Expression values relative to control (in %) of selected histone H3 modifications.

To better understand which functional related set of genes are affected by this early deregulation of the transcriptome upon treatment with Namodenoson, further downstream analysis was focused on the 682 DE genes at 24 hours (Table A2, Sheet 1). Gene set enrichment analysis^39^ identified a total of 155 differentially enriched gene sets with a padj value < 0.05 (Table A3). Four of them, targets of HDAC1, SUZ12, EZH2, and HDAC1/2 (Figure 3C) were associated with an epigenetic regulation pattern also involving HDACs, which alter transcription by deacetylating histones. This led us to hypothesize that ADORA3 stimulation might induce chromatin remodeling events in tumor cells. Next, we investigated the mRNA expression of the enzymes *HDAC1* to *HDAC 11* in HepG2 cells upon treatment with Namodenoson using RT-qPCR. Of note, all 11 HDACs were significantly downregulated after incubation with Namodenoson for 24 hours (Figure 3D). Subsequently, we performed a multiplexed analysis of the protein expression and modification status of 33 selected proteins and histone modification status with the high-throughput proteomic technique DigiWest^35^ to investigate a broader influence of Namodenoson on epigenetic factors. This analysis confirmed the downregulation of HDACs for the selected HDAC enzymes 1–3, and 6 (Figure 4A and B, Table A4A and B). Furthermore, this analysis revealed several histone H3 modifications, mainly including acetylation and methylation events, while the total levels of H3 remained unchanged (Figure 4A and C). Taken together, we discovered the regulation of epigenetic pathways as a new mode of action of the drug Namodenoson.

### Combination of ADORA3 Stimulation and HDAC Inhibition

**Figure 5:**
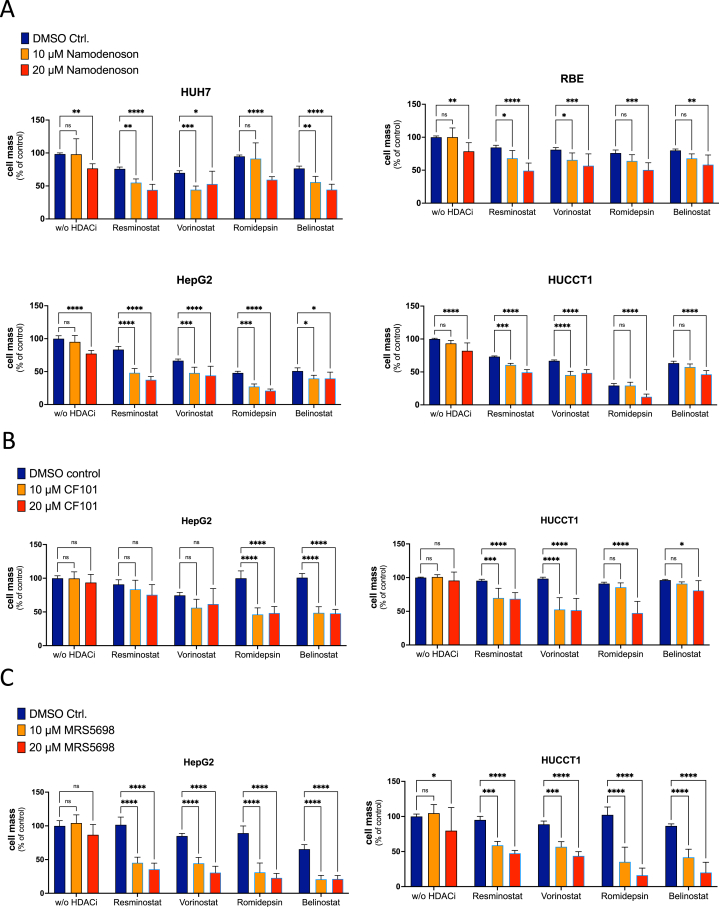
Combination treatment of different ADORA3 agonists and selected HDACis. Human-derived HCC cell lines HepG2 and Huh7 (left side), and CCA cell lines HUCCT1 and RBE (right side) were treated with ADORA3 agonists and 1 of 4 different drugs with HDACi activity for 72 hours. HDACi drugs were used with 1 μM Resminostat, 1 μM Vorinostat, 0.25 μM Belinostat, and 2 nM Romidepsin. SRB assays were performed to detect drug-induced cytotoxicities compared to controls. (A) Namodenoson (10 or 20 μM), (B) CF101 (5 or 10 μM), (C) MRS5698 (10 and 20 μM). Data represent standard error of the mean of 3 independent experiments. Statistical analysis was performed using 2-way ANOVA with Tukey’s multiple comparisons test (∗*P* < .05; ∗∗*P* .01, ∗∗∗*P* .001, ∗∗∗∗*P* < .0001, ns, not significant). SRB, Sulforhodamin B Cytotoxicity.

The observed pattern of downregulation of HDAC enzymes after Namodenoson treatment suggests a complementary downregulation compared to other drugs with HDAC inhibitory activity, especially HDACis. Therefore, HDAC inhibition in combination with stimulation of ADORA3 might be an attractive new combined drug therapy for the treatment of liver cancer. As a proof of concept, we chose 4 different HDACis that have either been tested in clinical trials with HCC patients (Resminostat, Belinostat, Vorinostat) or approved for the treatment of other tumor entities (Romidepsin). To examine whether the combination of Namodenoson and HDACi achieves synergistic effects on cell proliferation, 2 different hepatoma (Huh7 and HepG2) and CCA (RBE and HUCCT1) cell lines were treated with 1 of the 4 HDACis alone or in combination with 2 different concentrations of Namodenoson (10, 20 μM) for 72 hours. The concentration of the HDACi drugs was selected only to moderately inhibit proliferation, which was necessary to detect an additional effect by adding Namodenoson. As shown in Figure 5A, the addition of Namodenoson significantly enhanced the effect of any HDACi in almost all selected combinations. Notably, despite a varying degree of inhibition, this observation was not restricted to a single HDACi, suggesting that drugs with different HDACi activities are all candidates for combination treatment. Vice versa, to explore whether the combined effect is a unique feature of Namodenoson or can be regarded as a more general mechanism for ADORA3 activation, 2 additional ADORA3 agonists were investigated: the drug CF101 (Piclidenoson), which is in clinical testing for inflammatory disorders, and the chemical compound MRS5698. The treatment of HepG2 and HUCCT1 cells with Piclidenoson confirmed the inhibitory effect combined with several HDACi; however, the outcome seemed to be less prominent compared to the combinations with Namodenoson (Figure 5B). Triggering ADORA3 with MRS5698 demonstrated a profound inhibitory effect for all selected combinations (Figure 5C). In conclusion, these results suggest that the observed combination effect is a group effect of substances with ADORA3 agonistic and HDAC inhibitory activities.

### Stimulation of ADORA3 and Inhibition of HDACs in Patient-Derived HCC Organoids

**Figure 6:**
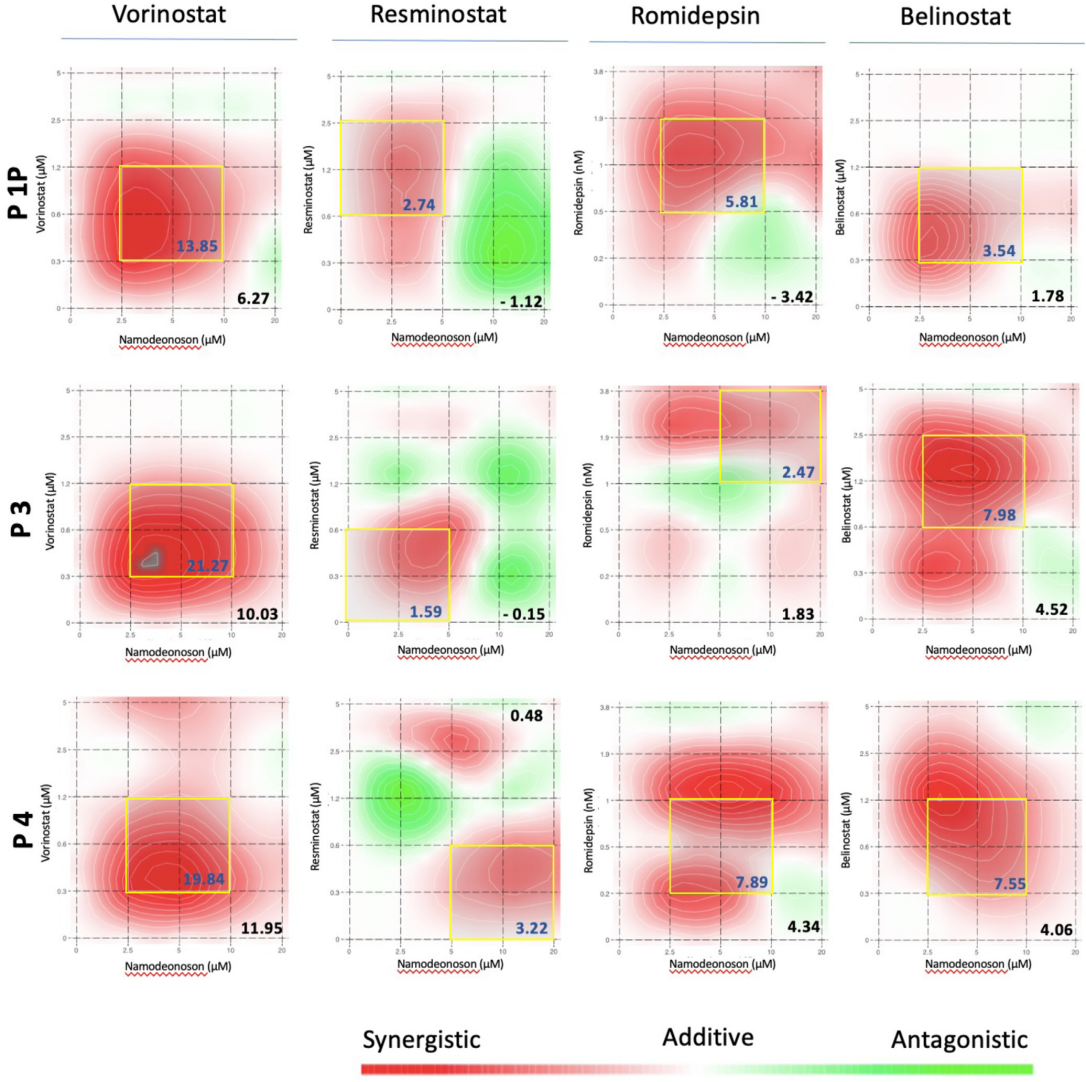
Namodenoson and HDACi combination therapy in patient-derived HCC organoid models. HCC organoids from 3 different patients (Pat. 1P, 3, 4) were investigated with the combination of Namodenoson and one of the 4 HDACi drugs Vorinostat, Resminostat, Romidepsin, and Belinostat. The drug combination interaction was investigated with SynergyFinder 2.0 to visualize and explore drug synergy landscapes.^37^ Namodenoson was investigated with 5 (20, 10, 5. 2.5, 0 μM) and the HDACi with 6 different concentrations (Vorinostat, Resminostat, and Belinostat with 5, 2.5, 1.25, 0.625, 0.312, 0 μM; Romidepsin with 3.8, 1.9, 0.95, 0.48, 0.24, 0 nM), resulting in 5 × 6 matrices to build and visualize synergy landscapes using a zero interaction potency reference model. After 6 days viability was evaluated using CellTiter-Glo 3D cell viability assay. The average synergy score is shown in the right lower corner of each graph, the most synergistic area and the corresponding synergistic area score is shown within a yellow square in each graph. Scores >10, the drug interactions are likely to be synergistic, 10 to −10, the interaction is likely to be additive, <−10, the interaction is likely to be antagonistic (https://synergyfinder.fimm.fi/synergy/synfin_docs/).

To explore the potential of a combination therapy with Namodenoson and HDACi in a human-derived cellular context, we finally investigated previously described organoids from tumor biopsies of 3 HCC patients (P1P, P3, P4).^30^ For these experiments, 5 different concentrations of Namodenoson and 6 different concentrations of the 4 previously described HDACi compounds were investigated. To detect a potential additive or synergistic effect of the combination therapies, we chose concentrations equal to or less than known IC_50_ values. The resulting 5 × 6 matrices were used to build synergy landscapes using a Zero interaction potency model with SynergyFinder 2.0.^36^^,^^37^ For each treatment combination, the *average synergy score* (shown in the right lower corner of each graph) and the *most synergistic area score* (within the yellow square of each graph) were calculated (Figure 6). As a result, all treatment combinations in all experimental settings reached an average synergy score in the range of an additive to synergistic interaction between −3.42 for P1P with Romidepsin and 11.95 for P4 with Vorinostat. The highest scores were found for the combination of Namodenoson and Vorinostat, followed by Belinostat, Romidepsin, and Resminostat. Of note, no average synergy score was below the threshold of −10 for likely antagonistic interactions between the 2 drugs. In summary, these experiments employing human-derived PDOs further suggest combining an ADORA3 agonist with an HDACi as a new option to target liver cancer.

## Discussion

Purinergic signals play an important role in liver homeostasis, tissue repair, or the crosstalk between liver-resident cells and recruited immune cells.^12^ In this context, alterations in ADORA signaling pathways occur during malignant transformation, which identifies these receptors, especially ADORA3, as a potential target to treat cancer.^9^^,^^10^^,^^16^^,^^17^^,^^40^^,^^41^ The first and so far only phase II study with HCC included patients with liver cirrhosis CHILD B without any stratification concerning ADORA3 due to the hypothesis that a general overexpression of ADORA3 receptors is present in HCC. However, this assumption has been based on one reported HCC patient sample with an increased signal in an RT-PCR analysis compared to tumor-adjacent liver tissue^8^ or a conclusion by analogy from colon or breast cancer tissues.^24^ To the best of our knowledge, our results are the first report of a detailed comparison of ADORA3 expression in HCC, CCA, and normal liver tissues. We found a profound ADORA3 expression in normal liver tissue by immunohistochemistry, as well as in public available (TCGA) mRNA expression data and own needle biopsy samples. Of note, we could not confirm higher ADORA3 levels in tumor tissues compared to normal liver tissues, neither for HCC nor CCA. Moreover, a substantial number of HCC and CCA samples revealed low or even missing ADORA3 signals in the immunohistochemistry analysis. As ADORA3 blockage by a receptor antagonist or siRNA investigation diminishes antiproliferative effects of Namodenoson (Figure 2D–F), these observations suggest that a certain level of ADORA3 expression might be necessary to employ a therapeutic ADORA3 stimulation successfully. Regarding the reported phase II study by Stemmer et al.,^26^ no information is available on ADORA3 expression, which might have helped to discriminate between responding and nonresponding patients.

While our study did not specifically focus on the differential effects of ADORA3 activation in normal vs cancerous liver cells, it is worth noting that clinical trials of Namodenoson in HCC patients^26^^,^^28^ or in metabolic dysfunction-associated steatotic liver disease and MASH^27^ have reported no hepatotoxicity. This suggests that normal hepatocytes and cholangiocytes may respond differently to ADORA3 agonists than cancer cells. This differential response could be attributed to distinct downstream signaling pathways or differences in the cellular microenvironment. Future research is warranted to further elucidate these mechanisms. Our findings highlight the importance of understanding ADORA3’s role not only in liver cancer but also in normal liver physiology, potentially guiding the optimization of ADORA3-targeted therapies.

Drug combinations in clinical oncology aim to target cancer cells at multiple levels to improve therapeutic efficacy and clinical outcome.^42^, ^43^, ^44^ Thus, besides the above-suggested consideration of ADORA3 expression levels in liver tumors, it is of interest to identify drugs suited for combination treatment. With this aim, we performed a transcriptome-wide analysis to get further insights into Namodenoson-induced antitumor effects and surprisingly found a profound altered gene expression pattern over time compared to untreated control cells. Interestingly, amongst those differentially expressed genes, Namodenoson triggered the alteration of chromatin-modifying factors including HDAC enzymes, which identifies epigenetic modulation as a newly discovered mode of action of Namodenoson.

Given the observation that Namodenoson downregulates a broad range of HDAC enzymes in tumor cells, we hypothesized that a combination of ADORA3 activation and inhibition of HDACs might be able to enhance the therapeutic efficacy of either treatment alone. To further explore this, we selected drugs with HDACi activity that have been either approved for the treatment of certain hematological malignancies (Vorinostat, Belinostat, Romidepsin) or that have already been investigated in early clinical studies for the treatment of liver cancer (Vorinostat,^45^ Belinostat,^46^ Resminostat^47^). First, all selected HDACi drugs enhanced the antiproliferative effect of Namodenoson to different degrees. Thus, HDAC inhibition can be regarded as a generalizable principle to improve ADORA3-directed anticancer therapy, seemingly independent of the individual inhibitory drug profile. However, the question of which HDACs should be inhibited to induce an ideal combination effect in different tumor types or genetic tumor backgrounds is beyond the scope of this investigation and should be elucidated in future studies. Vice versa, we investigated whether the combination of drugs with HDACi activity and ADORA3 activation is restricted to the drug Namodenoson or is expandable to other ADORA3 agonists. Of note, the structurally similar drug Piclidenoson (CF101) and the structurally different compound MRS5698 showed similar results with an even more prominent effect of MRS5698 compared to Piclidenoson. These results demonstrate that the observed effects of the combination therapy are not limited to single compounds but should be regarded as a group effect of drugs with ADORA3 activation and inhibition of HDACs.

Investigations in different mammalian species revealed marked differences in expression levels of ADORA3 within and between species, including interspecies differences in ligand recognition.^19^^,^^48^^,^^49^ Due to these variances, it is quite challenging to translate the results of ADORA3 signaling from animal models to human pathology or even therapeutic interventions.^18^^,^^41^ Thus, we tested Namodenoson and the 4 different HDACi in PDO models from 3 patients with HCC. These experiments additionally support an enhanced antitumoral effect of the combination therapy compared to monotherapies with the selected drugs alone. Of note, all employed drugs in the reported experiments are either approved for treating other malignancies or have already been applied in clinical studies of liver cancer, which means that the pharmacology and safety profile of these substances are well known. In conclusion, the further development of the concept of ADORA3 activation and inhibition of HDACs in a clinical study to treat liver cancer seems to be reasonable and promising, even without evidence of additional preclinical animal models. In such a scenario, the expression level of ADORA3 in tumor tissues might be an essential prerequisite for identifying patients that benefit from this approach due to our observation of quite variable ADORA3 levels in HCC- and CCA-derived patient samples.

## Supplementary Materials

Figures A1 and A2, and Tables A1, A4A–C, and A5

Supplementary Materials & Methods

Table A2 RNAseq analysis data of HCC derived HepG2 cells treated for 24 h with Namodenoson vs untreated control cells (DMSO, solvent control).

Table A3 Gene Set Enrichment Analysis (GSEA) of RNA-seq of HepG2 cells after the treatment with Namodenoson for 24 h.
