# Supplementary material for: Adenosine Receptor 3 in Liver Cancer: Expression Variability, Epigenetic Modulation, and Enhanced Histone Deacetylase Inhibitor Effects
Source: Gastro Hep Adv. 2024 Nov 20;4(3):100590. doi: 10.1016/j.gastha.2024.11.006 (PMC11795062; doi:10.1016/j.gastha.2024.11.006)
Supplement: Figures A1 and A2, and Tables A1, A4A–C, and A5 [file mmc1.pdf]

ADENOSINE RECEPTOR 3 IN LIVER CANCER: EXPRESSION VARIABILITY, EPIGENETIC MODULATION, AND ENHANCED HDAC INHIBITOR EFFECTS

Louise Kaldjob-Heinrich, Sandro Nuciforo, Steffen Lemke, Aaron Stahl, Stefan Czemmel, Sepideh Babaei, Lauriane Blukacz, Marie-Anne Meier, Yizheng Zhang, Christian M. Schürch, Irene Gonzalez-Menendez, Pascal Woelffing, Nisar P. Malek, Veit Scheble, Sven Nahnsen, Manfred Claassen, Markus Templin, Hans Bösmüller, Markus H. Heim, Daniel Dauch, Michael Bitzer

SUPPLEMENTARY FIGURES & TABLES

Table of Contents

Supplementary Figure 1	page 2
Supplementary Figure 2	page 3
Supplementary Table 1	page 4
Supplementary Table 4A	page 5
Supplementary Table 4B	page 6
Supplementary Table 4C	page 7
Supplementary Table 5	page 8

Supplementary Tables 2 and 3 are provided as seperate files in Ecxel format.

Supplementary Figure 1

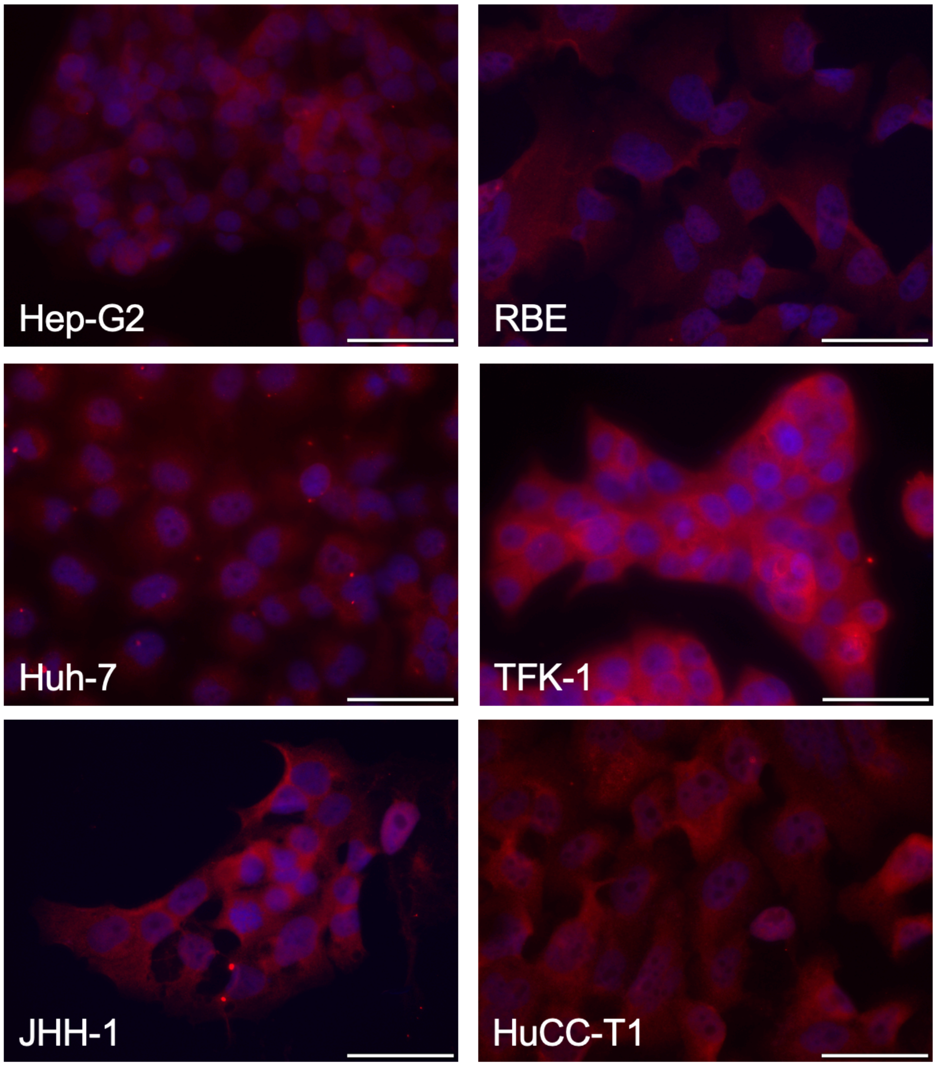

ADORA3 immunocytochemical staining in human-derived tumor cell lines.

Representative pictures of the human HCC cell lines Hep-G2, Huh-7 and JHH-1 as well as the CCA cell lines RBE, TFK-1 and HuCC-T1 upon immunocytochemical staining of Adora3 (red) and DAPI staining (blue). Scale bar = 50µm

Supplementary Figure 2

A

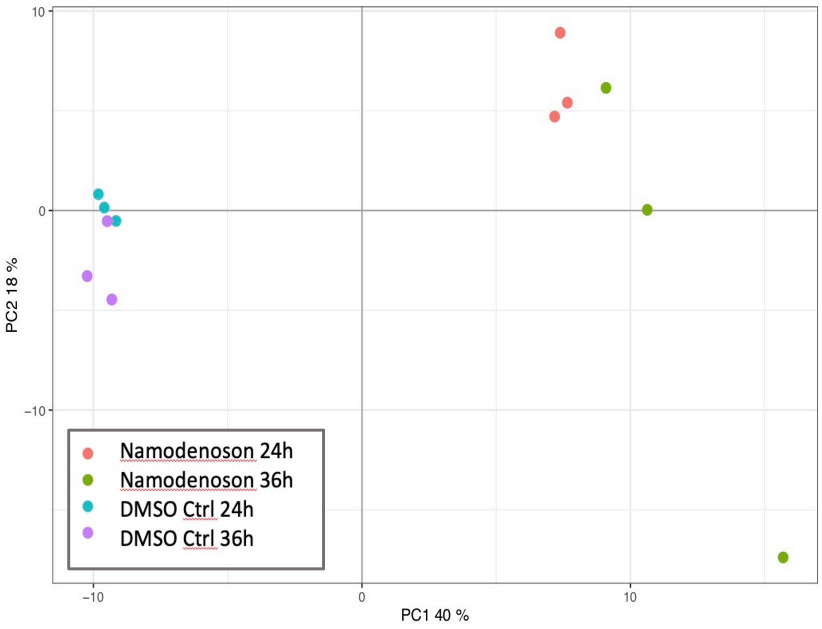

B

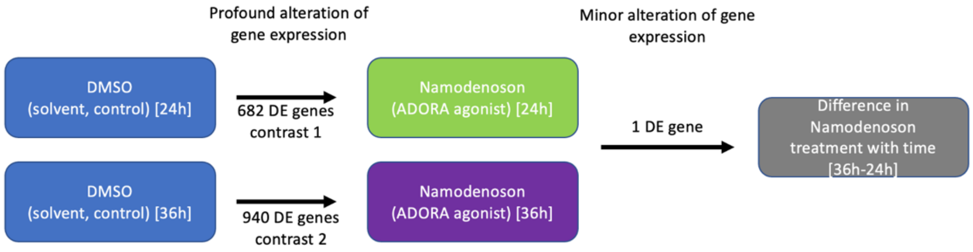

HepG2 RNA-Seq. analysis after treatment with Namodenoson.

(A) Principal component analysis (PCA) of the normalized RNA-seq data of all Namodenoson (ADORA3 agonist) and DMSO (solvent control) treated samples at 24 and 36 h. The percentage of variation explained by PC1 and PC2 is indicated at each axis. Colors indicate samples from different treatment and timepoint combinations.

(B) Number of genes with a significantly altered expression after treatment with Namodenoson for 24 h or 36 h compared to untreated control cells (DMSO, solvent control), and number of genes significantly altered by Namodenoson between 36h and 24h of treatment. Genes were considered differentially expressed when the adjusted p-value was lower than 0.05 (padj < 0.05) and a log2FC of greater than 0.5 or lower -0.5 was detected.

Supplementary Table 1

IC50 values and 95% confidence interval (95% CI) of Namodenoson treated (A) human-derived HCC and CCA cell lines and (B) patient-derived organoids.

A

IC50 Value Human HCC and CCA cell line			
HCC (95% CI)		CCA (95% CI)	
HepG2	35.3 (24.1 – 54.2)	HUCCT1	23.9 (17.3 – 33.5)
Huh7	31.8 (25.6 – 40.3)	RBE	46.8 (33.6 – 66.7)
JHH1	69.3 (38.9 – 177.5)	TFK1	13,9 (11.9 – 15.9)

B

IC50 Value Human HCC and CCA cell Organoids			
HCC (95% CI)		CCA (95% CI)	
Pat. 1	19.6 (17.2 – 22.2)	Pat.7	6.9 (3.3 – 13.8)
Pat.1P	15.2 (12.2 – 19.1)		
Pat. 2	17.8 (12.2 – 27.2)		
Pat. 2.2	15 (10.2 – 22.1)		
Pat. 3	6.3 (4.7 – 8.1)		
Pat. 4	10.5 (8.9 – 12.2)		
Pat. 5	12.1 (8.4 – 17.4)		
Pat. 6	18.1 (13.0 – 24.9)		

Supplementary Table 4A

DigiWest raw data (AFI) of control (n=2) and Namodenoson-treated (n=2) HepG2 cells.

Empty cell = weak signal (AFI = 33).

#	AFI	ctrl. 1	ctrl. 2	20µM Namo. 1	20µM Namo. 2
1	Bmi1	5633	5344	8494	8557
2	Ezh2	1655	1793	2387	2326
3	GCN5L2	578	655	712	781
4	Histone deacetylase 1 (HDAC1)	108	139		
5	Histone deacetylase 2 (HDAC2)	9891	10066	7972	8782
6	Histone deacetylase 3 (HDAC3)	65	101	60	
7	Histone deacetylase 6 (HDAC6)	3981	4220	3783	4945
8	Histone H2A.X - phospho_Ser139	114	58	122	224
9	Histone H2B	263131	133716	369729	409341
10	Histone H2B - acetyl_Lys15				58
11	Histone H2B - acetyl_Lys5	1723	1809	1850	2144
12	Histone H3	826404	657112	970060	918638
13	Histone H3 - acetyl_Lys14	108	122	187	301
14	Histone H3 - acetyl_Lys18	4765	3863	8694	13446
15	Histone H3 - acetyl_Lys23			2476	2937
16	Histone H3 - acetyl_Lys9	7281	4963	11358	12245
17	Histone H3 - acetyl_Lys9/Lys14	5394	3368	8704	8777
18	Histone H3 - dimethyl_Lys4	1069	577	1408	1750
19	Histone H3 - monomethyl_Lys4	996	568	1906	2247
20	Histone H3 - phospho_Ser10	294	132	64	145
21	Histone H3 - phospho_Thr3				
22	Histone H3 - trimethyl_Lys27	7896	4459	6795	7798
23	Histone H3 - trimethyl_Lys9	21432	13808	23637	24199
24	IDH1	5458	6034	8080	8650
25	IDH2	6340	6276	9193	9035
26	JARID1A		56		
27	PCAF (KAT2B)	61		76	72
28	PDI	1110	1183	2200	1896
29	RING1A	472	497	585	645
30	RING1B			80	68
31	SP1	117	166	64	54
32	SUZ12	558	614	729	689
33	TBP (TF2D)	1383	1006	1499	1291
-	total protein (strep-PE) 1	724525	631391	819697	934574
-	total protein (strep-PE) 2	727482	633040	821239	934672
-	total protein (strep-PE) 3	723749	631988	826719	940033

Supplementary Table 4B

DigiWest-based Log2 fold changes of respective Namodenoson-treated samples vs. mean control. Data were normalized to total protein signal (strep-PE).

#	Fold Change (Log2)	20µM Namo. 1	20µM Namo. 2
1	Bmi1	0,35	0,17
2	Ezh2	0,18	-0,04
3	GCN5L2	-0,08	-0,14
4	Histone deacetylase 1 (HDAC1)	-2,10	-2,10
5	Histone deacetylase 2 (HDAC2)	-0,61	-0,66
6	Histone deacetylase 3 (HDAC3)	-0,78	-1,53
7	Histone deacetylase 6 (HDAC6)	-0,40	-0,20
8	Histone H2A.X - phospho_Ser139	0,26	0,94
9	Histone H2B	0,65	0,61
10	Histone H2B - acetyl_Lys15	0,00	0,53
11	Histone H2B - acetyl_Lys5	-0,22	-0,19
12	Histone H3	0,11	-0,15
13	Histone H3 - acetyl_Lys14	0,41	0,91
14	Histone H3 - acetyl_Lys18	0,74	1,18
15	Histone H3 - acetyl_Lys23	6,13	6,19
16	Histone H3 - acetyl_Lys9	0,63	0,55
17	Histone H3 - acetyl_Lys9/Lys14	0,73	0,55
18	Histone H3 - dimethyl_Lys4	0,52	0,65
19	Histone H3 - monomethyl_Lys4	1,03	1,08
20	Histone H3 - phospho_Ser10	-1,97	-0,99
21	Histone H3 - phospho_Thr3	0,00	0,00
22	Histone H3 - trimethyl_Lys27	-0,12	-0,11
23	Histone H3 - trimethyl_Lys9	0,16	0,01
24	IDH1	0,20	0,11
25	IDH2	0,26	0,05
26	JARID1A	-0,60	-0,60
27	PCAF (KAT2B)	0,55	0,27
28	PDI	0,65	0,25
29	RING1A	-0,02	-0,06
30	RING1B	1,17	0,74
31	SP1	-1,44	-1,89
32	SUZ12	0,03	-0,24
33	TBP (TF2D)	0,06	-0,34

Supplementary Table 4C

Antibody list for DigiWest.

#	Antigen	Mod-Site(s)	Supplier	Product No.	Species	MW [kDa]
1	Bmi1		Cell Signaling	6964	rb	43, 41
2	Ezh2		Cell Signaling	5246S	rb	98
3	GCN5L2		Cell Signaling	3305	rb	94
4	Histone deacetylase 1 (HDAC1)		Cell Signaling	2062	rb	62
5	Histone deacetylase 2 (HDAC2)		Epitomics	1603-1	rb	55
6	Histone deacetylase 3 (HDAC3)		Epitomics	1580-1	rb	49
7	Histone deacetylase 6 (HDAC6)		Millipore	07-732	rb	134
8	Histone H2A.X - phospho	Ser139	Cell Signaling	9718	rb	15
9	Histone H2B		Cell Signaling	12364	rb	14
10	Histone H2B - acetyl	Lys15	Epitomics	2170-1	rb	17
11	Histone H2B - acetyl	Lys5	Millipore	07-382	rb	16-14
12	Histone H3		abcam	ab1791	rb	17
13	Histone H3 - acetyl	Lys14	Millipore	17-10051	rb	17
14	Histone H3 - acetyl	Lys18	Cell Signaling	9675	rb	17
15	Histone H3 - acetyl	Lys23	Cell Signaling	8848	rb	17
16	Histone H3 - acetyl	Lys9	Cell Signaling	9649	rb	17
17	Histone H3 - acetyl	Lys9/Lys14	Calbiochem	382158	rb	17
18	Histone H3 - dimethyl	Lys4	Epitomics	1347-1	rb	17
19	Histone H3 - monomethyl	Lys4	Cell Signaling	5326	rb	17
20	Histone H3 - phospho	Ser10	Cell Signaling	9701	rb	17
21	Histone H3 - phospho	Thr3	Millipore	17-10141	rb	17
22	Histone H3 - trimethyl	Lys27	Cell Signaling	9756	rb	17
23	Histone H3 - trimethyl	Lys9	Millipore	07-523	rb	17
24	IDH1		Cell Signaling	8137	rb	46
25	IDH2		abcam	ab55271	ms	50
26	JARID1A (H3 K4-demethylase)		Cell Signaling	3876	rb	200
27	PCAF (KAT2B)		Cell Signaling	3378	rb	93
28	PDI		Cell Signaling	3501	rb	57
29	RING1A		Cell Signaling	2820	rb	58
30	RING1B		Cell Signaling	5694	rb	41
31	SP1		Cell Signaling	9389	rb	90
32	SUZ12		Cell Signaling	3737	rb	83
33	TBP (TF2D)		Cell Signaling	8515	rb	38

Supplementary Table 5

Primers for quantitative real-time qRT-PCR for mRNA quantification.

Oligo Name	Sequence (5'- 3')	Reference / designed by
**HDAC1-F**	CATCTCCTCAGCATTGGCTT	Tao et al.
**HDAC1-R**	CGAATCCGCATGACTCATAA	Tao et al.
**HDAC2-F**	ATGAGGCTTCATGGGATGAC	Tao et al.
**HDAC2-R**	ATGGCGTACAGTCAAGGAGG	Tao et al.
**HDAC3-F**	CTGTGTAACGCGAGCAGAAC	Tao et al.
**HDAC3-R**	GCAAGGCTTCACCAAGAGTC	Tao et al.
**HDAC4-F**	CTGGTCTCGGCCAGAAAGT	Tao et al.
**HDAC4-R**	CGTGGAAATTTTGAGCCATT	Tao et al.
**HDAC5-F**	GAACTGGGCATGGCTCTTG	Tao et al.
**HDAC5-R**	GGGAACCATCCTTGGAAATC	Tao et al.
**HDAC6-F**	GCGGTGGATGGAGAAATAGA	Tao et al.
**HDAC6-R**	CCGGAGGGTCCTTATCGTAG	Tao et al.
**HDAC7-F**	CCTGCTGTTGTCACCGC	Saha et al.
**HDAC7-R**	TCCTCTCCAGCTCAGAGACC	Saha et al.
**HDAC8-F**	GCGTGATTTCCAGCACATAA	Tao et al.
**HDAC8-R**	ATACTTGACCGGGGTCATCC	Tao et al.
**HDAC9-F**	GCCCACAGGAACTTCTGACT	Tao et al.
**HDAC9-R**	GAACTCTAAGCCAGATGGGG	Tao et al.
**HDAC10-F**	GAACAGCCACATCCAGGG	Tao et al.
**HDAC10-R**	CCTCTTAGATGGGATGCTGG	Tao et al.
**HDAC11-F**	AAGGAAGTTGGGGAGGAAGA	Tao et al.
**HDAC11-R**	GCACACGAGGCGCTATCTTA	Tao et al.

References

Tao, Y.-F., et al., Differential mRNA expression levels of human histone-modifying enzymes in normal karyotype B cell pediatric acute lymphoblastic leukemia. Int J Mol Sci, 2013. 14(2): p. 3376-3394.

Saha, A., et al., Epigenetic silencing of tumor suppressor genes during in vitro Epstein–Barr virus infection. Proc Natl Acad Sci USA, 2015. 112(37): p. E5199.
