# Supplementary material for: Adenosine Receptor 3 in Liver Cancer: Expression Variability, Epigenetic Modulation, and Enhanced Histone Deacetylase Inhibitor Effects
Source: Gastro Hep Adv. 2024 Nov 20;4(3):100590. doi: 10.1016/j.gastha.2024.11.006 (PMC11795062; doi:10.1016/j.gastha.2024.11.006)
Supplement: Supplementary Materials & Methods [file mmc2.pdf]

Supplementary Material & Methods

Substances. ADORA 3 receptor agonists: Namodenoson (Cat. No. HY12365, Hycultec), Piclidenoson (CF101, Cat. No. 1016; Tocris) MRS5698 (Cat. No. 5428; Tocris) and ADORA 3 receptor antagonist: MRE3008F20 (Cat. No. HY103178). Histone deacetylase inhibitors (HDACis): Resminostat (HY-14718A), Romidepsin (Cat. No. HY-15149), Belinostat (Cat. No. HY-10225), Vorinostat (Cat. No. HY-10221) were purchased from Hycultec. All substances were dissolved in DMSO at 10 mM aliquots, and stored at 20°C.

Cell Culture and human HCC and CCA organoid cultures. Human-derived cell lines HepG2 (ACC 180), and TFK1 (ACC 344) were obtained from the German Collection of Microorganisms and Cell Cultures (DSMZ, Braunschweig, Germany). HUCCT1 (RCB1960, RRID:CVCL_0324), RBE (RCB1292), and HuH-7 (RCB1366, RRID:CVCL_0336) were purchased from Riken Cell Bank (Tokyo, Japan), JHH1 from the Japanese Collection of Research Bioresources (JCRB) Cell Bank. HepG2, Huh7, HUCCT1, RBE and JHH1 were cultured in Dulbecco's modified Eagle medium (DMEM, Sigma-Aldrich, Darmstadt, Germany) with 10% fetal bovine serum (FBS, Sigma-Aldrich, Darmstadt, Germany) TFK1 cells were cultured in RPMI-1640 medium containing 10% FBS. All cells were cultured in a humidified incubator (37°C, 5% CO2) and tested regularly to be negative for mycoplasma contamination.

Cell viability assays. Established cell lines were plated in 24-well plates (3 x 10^4 cells / well) and allowed to adhere for 24 h. After the medium was changed, the cells were incubated with the indicated substances and controls, which were prepared in DMEM. Drug-induced cytotoxicities were determined after an incubation time of 72 h, employing the Sulforhodamine B assay (SRB, measuring remaining cell mass) [1, 2]. Cells were fixed with cold 10% trichloroacetic acid (TCA, Cat. No. T6399, Sigma) and dried at 55°C. Cells were stained with SRB staining solution (0.4%

Sulforhodamine B; HY-D0974, Hycultec, in 1% acetic acid; Cat. No. 1000631011, Sigma), and after drying overnight, the stain was solubilized in 10 mM Tris base, pH 10.5. Optical density was measured at a wavelength of 550 nm. Differences in cell mass were obtained by comparing DMSO treated control to drug treated wells with controls set to 100%. Results are shown as mean ±SEM.

Viability of PDOs was measured using CellTiter-Glo 3D reagent (Promega). In brief, single cell suspensions of tumor organoids were plated in a 384-well plate at a density of 1000 cells / well. After two days of culture to allow recovery and formation of 3D structure, the drug solution or DMSO solvent controls were added. After 6 days of treatment cell viability was measured, following the provider´s instructions. Luminescence was measured on a Synergy H1 Multi-Mode Reader (BioTek Instruments). Results were normalized to the solvent (100% DMSO). Curve fitting was performed using Prism (GraphPad Prism; RRID:SCR_002798)) software and the nonlinear regression equation. All experiments were performed at least two times in duplicate. Results are shown as mean ±SEM.

siRNAs transfection. HepG2 and HUCCT1 cells ($4x10^5$) were seeded in 6-well plates and grown up to 70% confluence. After 24 h, cells were transfected by using Silencer® Select with ADORA3 siRNA (Cat. AM16708, ThermoFischer Scientific), non-target siRNA (Cat. 4390844, ThermoFischer Scientific) and Lipofectamine 3000 reagent (Cat. No. L3000015, ThemoFisher Scientific) according to the manufacturer's instructions. Briefly, 7.5 µl Polyfectamine 3000 reagent per well was diluted with 250 µl of serum-free medium for 10 min at room temperature. 100 µM siRNA stock was generated by adding 50 µl RNAse-free H20 to 5 nmol siRNA. Final concentration of 800 nM siRNA per well was used. siRNA mastermix was prepared by diluting siRNA to serum-free medium and P3000^{TM} Reagent (250 µl/well). Polyfectamine solution (250 µl) was added to diluted siRNAs. After incubation for 15 min at room temperature, the DNA-lipid complex was

added to each well containing 0.5 ml of culture medium with 10% FBS, resulting in 1 ml of total transfection volume. After 48 h of incubation at 37°C medium was changed, and transfected cells were treated accordingly for another 72 h.

Tissue microarray (TMA). For each case, a total of 3 cores representing tumor tissue and 3 cores with normal liver tissue were investigated. For immunohistochemistry, the primary antibody ADORA3 from Signalway Antibody (SAB, Cat. No. 35615, Baltimore, USA) was applied. Tissue sections were pretreated with Tris-based buffer solution (slightly basic pH) at 100°C for 64 min. The detection kit required for staining was OptiView DAB (Roche, Mannheim, Germany). For quantification of ADORA3 expression, immunohistochemistry slides were scanned using Pannoramic Midi II (3DHISTECH Ltd. Budapest, Hungary). Tissue microarray image processing was performed on QuPath software [3]. TMA cores were detected by the TMADearrayer tool and DAB staining intensity per pixel was computed. Visualization and statistics were performed using GraphPad Prism software (GraphPad Inc., San Diego, CA, USA).

Quantitative real-time qRT-PCR for mRNA quantification. Total RNA was isolated from cells using the NucleoSpin® RNA purification Kit (Macherey-Nagel, Düren, Germany) following manufacturer's instructions. 1 µg of RNA was reversed transcribed to complementary-DNA using the QuantiTect Reverse Transcription Kit (QIAGEN, Hilden, Germany) following manufacturer's instructions. Selected genes were amplified and analyzed using the corresponding oligo (dt) primers, a SYBR™ Select Master Mix (ThermoFisher Scientific) and the LightCycler 480 System (Roche). Amplification was performed after a 10 min hold stage at 95°C and 40 cycles of denaturation at 95°C for 15 s followed by a 1 min annealing and elongation phase at 60°C. The melting curve data were collected to check primer quality and specificity. A detailed list of all used primers can be found in Suppl. Tab. 5. Real time polymerase chain reaction (qPCR) results were

presented relative to the mean of GAPDH ($2^{\Delta\Delta Ct}$ method). Each cDNA sample was analyzed as triplicate and corresponding samples with no cDNAs were included as negative controls [4, 5].

RNA-Seq Analysis and Bioinformatics. The Nextflow-based nf-core RNA-Seq pipeline release 1.3 (https://github.com/nf-core/rnaseq1) was used for the bioinfomatics analysis. An aggregation of the bioinformatics workflow analysis was conducted by MultiQC v1.72 (RRID:SCR_014982) [6]. FASTQC3 [7] was used to determine quality of the FASTQ files. Subsequently, adapter trimming was conducted with Trim Galore v0.5.04 (RRID:SCR_011847) [8]. STAR v2.6.1d (RRID:SCR_004463) [9] aligner was used to map the reads that passed the quality control to the human genome GRCh 38 (Ensembl release 92). The evaluation of the RNA-seq experiment was performed with RSeQC v3.0.0 (RRID:SCR_005275) [10] and read counting of the features (e.g. genes) was done with featureCounts v1.6.4 (RRID:SCR_012919) [11] For differential expression analysis, the raw read count table resulting from featureCounts was processed with the nextflow-based workflow qbic-pipelines/rnadeseq version 1.3.2 (https://github.com/qbic-pipelines/rnadeseq/releases/tag/1.3.2) mainly utilizing the R package DESeq2 v1.22.1 (RRID:SCR_000154) [12] using R version 3.5.1 (2018-07-02). Graphs were produced mainly using the R package ggplot2 v3.3.0 (RRID:SCR_014601). Final reports were produced using the R package rmarkdown v2.1, with knitr v1.28 and DT v0.13 R packages. For the differential expression analysis of the TCGA data, the R package DESeq2 v1.30.1 with R version 4.0.3 was used outside of above mentioned workflow. The p-values were corrected for multiple testing using the Benjamini-Hochberg method [https://doi.org/10.1111/j.2517-6161.1995.tb02031.x]. For the plots, the gene counts were converted to transcript per million (TPM) values by utilizing the median transcript length for each gene, determined using GTFtools [https://doi.org/10.1101/263517].

TCGA-LIHC RNA-Seq tissue biopsy data. For the differential expression analysis, the R package DESeq2 v1.30.1 with R version 4.0.3 was used. The p-values were corrected for multiple testing using the Benjamini-Hochberg method [13]. For the plots, the gene counts were converted to transcript per million (TPM) values by utilizing the median transcript length for each gene, determined using GTFtools [14]. The plots were created using the Python package seaborn v0.10.1 (RRID:SCR_018132) with Python version 3.8.3.

Gene set enrichment analysis (GSEA, RRID:SCR_003199**).** GSEA was used to identify functional enrichment by comparing genes of interest with predefined gene sets. A gene set is defined as a group of genes that shares localization, pathways, functions, or other features. Gene set enrichment analysis (GSEA) was performed using the Bioconductor package fgsea (RRID:SCR_006442) [15]. All human gene sets from Molecular Signatures Database (MSigDB) gene sets [16] were examined. The fdr-adjusted p-value<0.05 was used to select statistically significant gene sets.

Immunoblotting. Cells were lysed on ice with 100 µl RIPA lysis buffer (50 mM Tris, 150 mM NaCl, 2mM EDTA, 1% Triton X-100, 0.1% SDS, pH to 7.8 supplemented with 0.1% Halt™ protease and phosphatase inhibitor cocktail (Cat. No. 78444, ThermoFisher Scientific) and 0.1% Phenylmethylsulfonyl fluoride (PMSF, Cat. No. 10837091001, Sigma/Merck)) per well. Cell lysates were incubated on a rotating wheel for 1 h at 4°C after two rounds of 5 sec sonication (Bandelin Sonoplus) at a frequency of 20 kHz. Lysates were thereafter centrifuged at 12,000 g (7 min, 4 °C). The supernatants were used for Western blot analysis. Protein concentration was determined with the Pierce BCA Protein Assay Kit (Cat. No. 23227 ThermoFisher Scientific) using BSA as a standard at different concentrations to generate a regression curve. At least 15 µg of protein was mixed with NuPage sample buffer (Cat. No. NP0008, ThermoFischer Scientific) supplemented with 50 µM DTT. Protein lysates were boiled at 94°C for 5 min. 15 to 40 µg of

protein were loaded onto 4-12% SDS-PAGE gels and then transferred to polyvinylidene difluoride (PVDF) membranes (Millipore). After blotting membranes were blocked with 5% nonfat milk in Tris-buffered saline-Tween (TBST) for 1 h and incubated with the diluted primary antibodies ADRORA3 (Sygnalway Antibodies) or β-actin (Cell signalling) at 4°C overnight. Membranes were incubated with appropriate HRP conjugated secondary antibodies (BioRad) for 1 h the next day. An ECL chemiluminescence detection kit (BioRad, USA) was used to detect signals in accordance with the manufacturer's recommendations. ImageJ (RRID:SCR_003070) was used to quantify the integrated density of the band.

Immunocytochemical ADORA3 staining. Immunocytochemical staining of human liver cancer cells was performed on tissue culture chambers (Sarstedt). The cells were fixed with 4% paraformaldehyde, blocked with 5% BSA and stained with an antibody specific for Adora3 (Alomone Labs, AAR-004, Dilution 1:100) and DAPI (Vector Labs). Microscopic analyses were performed using the Olympus BX63 microscope (cellSens Dimension v1.17). Pictures were analyzed using the OlyVIA 3.2.1 software (Olympus).

DigiWest Method

Lysis and protein quantification. Cells were lysed on ice using 80-100 µl of 2x LDS Lysis Buffer (Life Technologies, Carlsbad, CA, USA), supplemented with a sample reducing agent (Thermo Fisher Scientific, Waltham, MA, USA), Protease-and Phosphatase Inhibitor (Roche Diagnostics GmbH, Mannheim, Germany). Proteins were denatured at 95°C for 10 mins before the lysates were transferred to QiaShredder tubes (Eppendorf, Hamburg, Germany). After centrifugation protein quantification was performed using in-gel staining. 1 µl of each original lysate was diluted in lysis buffer, and 10 µL were run in a NuPAGE 4-12% Bis-Tris precast gel (Thermo Fisher Scientific). All separated proteins were stained with BlueBandit (VWR, Darmstadt, Germany) for

1 h. After de-staining, detection was performed on a LI-COR (LI-COR, Bad Homburg, Germany) instrument. Quantification was performed using ImageStudio.

For DigiWest, 12 µg of cellular protein were loaded on an SDS- polyacrylamide gel and size-separated using the commercial NuPAGE system (Life Technologies). Size-separated proteins were blotted onto a PVDF membrane and biotinylated on the membrane using NHS-PEG12-Biotin (50 µM) in PBST for 1 h. After drying of the membrane, the samples lanes were cut into 96 strips of 0.5 mm width using an automated cutting plotter (Silhouette America, West Orem, UT, USA) each corresponding to a defined molecular weight fraction. Each of the strips was placed in one well of a 96-well plate and 10 µl elution buffer (8 M urea, 1% Triton-X100 in 100 mM Tris-HCl pH 9.5) was added. The eluted proteins were diluted with 90 µl of dilution buffer (5% BSA in PBS, 0.02% sodium azide, 0.05% Tween-20) and each of the protein fractions was incubated with 1 distinct magnetic color-coded bead population (Luminex, Austin, USA) coated with neutravidin. The biotinylated proteins bind to the neutravidin beads such that each bead color represents proteins of one specific molecular weight fraction. All 96 protein loaded bead populations were mixed resulting in reconstitution of the original lane. Such a bead-mix was sufficient for about 150 individual antibody incubations. Aliquots of the DigiWest bead-mixes (about 1/200th per well) were added to 96 well plates containing 50 µl assay buffer (Blocking Reagent for ELISA (Roche, Rotkreuz, Switzerland) supplemented with 0.2% milk powder, 0.05% Tween-20 and 0.02% sodium azide) and different diluted primary antibodies were added to the wells. A complete list of all antibodies used for DigiWest can be found in supplementary table X. After overnight incubation at 15°C in a shaker, the bead-mixes were washed twice with PBST and species-specific PE-labelled (Phycoerythrin) secondary antibodies (Dianova, Hamburg, Germany) were added and incubated for 1 h at 23°C. Beads were washed twice prior to readout on a Luminex FlexMAP 3D. For quantification of the antibody-specific signals, an Excel-based analysis tool was employed [17] that automatically identifies peaks of appropriate molecular weight and calculates the peak area (reported as accumulated fluorescence intensity = AFI). Signal intensity was normalized to the

total amount of protein loaded onto one lane. HCL was performed using Pearson Correlation and complete linkage.

Statistical Analysis.

P value calculations outside of RNA-Seq and Gene Set Enrichment Analysis (GSEA) analysis were calculated with a One-way analysis of variance with Bonferroni's multiple comparison test when comparing multiple groups. All analyses were performed using GraphPad Prism v.8.0 (RRID:SCR_002798). Results in the figures are expressed as mean values with their standard errors. According to this analyses, P values > .05 were marked as not significant (n.s.) and P values < .05 were considered to indicate a statistically significant difference.

References

1. Skehan P, Storeng R, Scudiero D, et al. New colorimetric cytotoxicity assay for anticancer-drug screening. J Natl Cancer Inst 1990;82:1107-12.
2. Vichai V, Kirtikara K. Sulforhodamine B colorimetric assay for cytotoxicity screening. Nat Protoc 2006;1:1112-6.
3. Bankhead P, Loughrey MB, Fernandez JA, et al. QuPath: Open source software for digital pathology image analysis. Sci Rep 2017;7:16878.
4. Livak KJ, Schmittgen TD. Analysis of relative gene expression data using real-time quantitative PCR and the 2(-Delta Delta C(T)) Method. Methods 2001;25:402-8.
5. Higuchi R, Fockler C, Dollinger G, et al. Kinetic PCR analysis: real-time monitoring of DNA amplification reactions. Biotechnology (N Y) 1993;11:1026-30.
6. Ewels P, Magnusson M, Lundin S, et al. MultiQC: summarize analysis results for multiple tools and samples in a single report. Bioinformatics (Oxford, England) 2016;32:3047-3048.
7. Andrews S. FastQC: A Quality Control Tool for High Throughput Sequence Data. Available online at: http://www.bioinformatics.babraham.ac.uk/projects/fastqc/ 2010.
8. Krueger F. Trim Galore: A Wrapper Tool Around Cutadapt and Fastqc to Consistently Apply Quality and Adapter Trimming to Fastq Files, with Some Extra Functionality for Mspi-Digested Rrbs-Type (Reduced Representation Bisufite-Seq) Libraries. URL Http://Www. Bioinformatics. Babraham. Ac. Uk/Projects/Trim_galore/.(Date of Access: 28/04/2016). 2012.
9. Dobin A, Davis CA, Schlesinger F, et al. STAR: ultrafast universal RNA-seq aligner. Bioinformatics 2013;29:15-21.
10. Wang L, Wang S, Li W. RSeQC: quality control of RNA-seq experiments. Bioinformatics 2012;28:2184-5.
11. Liao Y, Smyth GK, Shi W. featureCounts: an efficient general purpose program for assigning sequence reads to genomic features. Bioinformatics 2014;30:923-30.
12. Love M, Anders S, Huber W. Differential analysis of count data@ the DESeq 2 package, 2014.
13. Benjamini Y, Hochberg Y. Controlling the False Discovery Rate - a Practical and Powerful Approach to Multiple Testing. Journal of the Royal Statistical Society Series B-Statistical Methodology 1995;57:289-300.
14. Li H-D. GTFtools: a Python package for analyzing various modes of gene models. bioRxiv 2018:263517.
15. Korotkevich G, Sukhov V, Budin N, et al. Fast gene set enrichment analysis. bioRxiv 2021:060012.
16. Liberzon A, Birger C, Thorvaldsdóttir H, et al. The Molecular Signatures Database (MSigDB) hallmark gene set collection. Cell Syst 2015;1:417-425.
17. Treindl F, Ruprecht B, Beiter Y, et al. A bead-based western for high-throughput cellular signal transduction analyses. Nat Commun 2016;7:12852.
